# The Interplay among Radiation Therapy, Antibiotics and the Microbiota: Impact on Cancer Treatment Outcomes

**DOI:** 10.3390/antibiotics11030331

**Published:** 2022-03-02

**Authors:** Kavery Nivana Theethira Poonacha, Tomás G. Villa, Vicente Notario

**Affiliations:** 1 George Squared Program, College of Science, George Mason University, Manassas, VA 20110, USA; kpoonach@gmu.edu; 2 Department of Microbiology, Faculty of Pharmacy, University of Santiago de Compostela, Santiago de Compostela, 15705 La Coruña, Spain; tomas.gonzalez@usc.es; 3 Department of Radiation Medicine, Georgetown University Medical Center, Washington, DC 20057, USA

**Keywords:** antibiotics, adverse events, cancer, dysbiosis, microbiota, radiation-induced toxicity, radiation therapy, radiosensitization, patient treatment outcomes

## Abstract

Radiation therapy has been used for more than a century, either alone or in combination with other therapeutic modalities, to treat most types of cancer. On average, radiation therapy is included in the treatment plans for over 50% of all cancer patients, and it is estimated to contribute to about 40% of curative protocols, a success rate that may reach 90%, or higher, for certain tumor types, particularly on patients diagnosed at early disease stages. A growing body of research provides solid support for the existence of bidirectional interaction between radiation exposure and the human microbiota. Radiation treatment causes quantitative and qualitative changes in the gut microbiota composition, often leading to an increased abundance of potentially hazardous or pathogenic microbes and a concomitant decrease in commensal bacteria. In turn, the resulting dysbiotic microbiota becomes an important contributor to worsen the adverse events caused in patients by the inflammatory process triggered by the radiation treatment and a significant determinant of the radiation therapy anti-tumor effectiveness. Antibiotics, which are frequently included as prophylactic agents in cancer treatment protocols to prevent patient infections, may affect the radiation/microbiota interaction through mechanisms involving both their antimicrobial activity, as a mediator of microbiota imbalances, and their dual capacity to act as pro- or anti-tumorigenic effectors and, consequently, as critical determinants of radiation therapy outcomes. In this scenario, it becomes important to introduce the use of probiotics and/or other agents that may stabilize the healthy microbiota before patients are exposed to radiation. Ultimately, newly developed methodologies may facilitate performing personalized microbiota screenings on patients before radiation therapy as an accurate way to identify which antibiotics may be used, if needed, and to inform the overall treatment planning. This review examines currently available data on these issues from the perspective of improving radiation therapy outcomes.

## 1. Introduction

The last few years of the nineteenth century (1895–1900) provided the scientific community with fundamental findings that became extraordinarily important for the later development of radiation-based clinical applications. In this regard, the initial discovery of radioactivity by Henri Becquerel and the identification and characterization of various radioactive minerals by Marie and Pierre Curie were undoubtedly important events. However, it was the discovery of the X-rays by Wilhelm C. Roentgen in 1895 and the subsequent establishment of procedures for using X-ray sources in a controlled way that opened the door to the use of ionizing radiation (IR) for clinical purposes [1,2]. Early oncological applications focused on exploiting IR for cancer diagnosis and anti-cancer therapy. However, the initial optimism brought about by the recognition that daily radiation doses over several weeks improved the cure chances of cancer patients was quickly tempered by the realization that IR exposure could also promote the onset and development of tumors and that, therefore, its clinical applications needed to be tightly regulated. Nevertheless, such initial negative perception provided the impetus for investigations on several important areas of research that greatly expanded our knowledge of radiation biology. Most importantly, it led to fundamental advances for the development of external beam-based radiation therapy (RT) protocols for the treatment of cancer, including (a) the use of different IR sources, (b) progress in studies on radiation physics, (c) incorporation of advanced computer technologies to the treatment planning, and (d) technologically advanced equipment that facilitates the precise delivery of radiation to the tumors with minimal damage to the normal tissues [3,4]. Although protocols for the internal delivery of radiation to tumors such as brachytherapy [5,6,7], the localized implant of radioactive seeds into, or as near as possible to, the tumor tissues, as well as the systemic treatment with tumor-specific, receptor-targeted radiopharmaceuticals [8,9,10], have become valuable and effective alternatives or complements to external-beam RT, this review will focus on the influence of the microbiota and the use of antibiotics on cancer patient outcomes after treatment with external-beam RT.

Despite the RT advances described above, exposure of cancer patients to IR may still cause various levels of clinical adverse events. The severity of RT toxicity depends mainly on the IR dose received and the tissue/organ targeted [11,12]. Two main mammalian organs have been identified as IR susceptible targets and, therefore, as significant contributors to the induction of RT-mediated adverse events and the overall response of patients to IR toxicity. The first one is the gut, an anatomical organ characterized as being particularly radiosensitive [11,13,14,15,16]. The second is the microbiota, particularly the gut microbiota, the trillions of commensal microbes (bacteria, archaea, fungi, and even bacteriophages) that live in the gut and maintain a symbiotic interaction with the host, jointly behaving as a “functional organ” that plays essential roles in balancing homeostatic processes to preserve normal physiological pathways in healthy individuals [17,18]. It is, therefore, important to point out that when RT is used to treat cancer patients, their microbiota is also exposed to IR. It is even more important to highlight the fact that in the case of a majority of cancer patients, their microbiota is already different from the normal gut microbiota of individuals free of cancer. In addition to already reported quantitative and qualitative differences in the gut microbiota between cancer patients and normal individuals, it has been well established by now that there is also a tumor-specific microbiota that is primarily comprised of intracellular microbes that can be detected at various frequencies in diverse cancer types [19,20,21].

The various microbial species that constitute the microbiota have different intrinsic radiosensitivities. Consequently, radiation exposure alters both the composition and the relative proportions of microbial populations, particularly in the gut. Typically, exposure to IR results in the time- and dose-dependent increase in *Firmicutes* and intracellular symbionts, with a concomitant decrease in *Bacteroidetes* species [22,23]. Overall, IR exposure leads to microbiota dysbiosis, resulting in a relative reduction in beneficial microbes such as *Bifidobacterium* and *Faecalibacterium* and a parallel increase in harmful or even pathogenic microorganisms such as *Fusobacteria* and *Proteobacteria*. The effect of RT on the microbiota could be exerted through a direct action on the microbes themselves, particularly lowering the proportion of those secreting beneficial short-chain fatty acids (SCFAs) such as acetate, butyrate, propionate, and others [24,25]. In addition, RT may also indirectly affect the microbiota by acting on the intestinal tissues, mainly when RT is used to treat abdominal and pelvic malignancies. In these cases, RT causes intestinal injuries that create an imbalance between the microbiota and the host that may favor the colonization by undesirable microbes, break the intestinal epithelial barrier (IEB), promote food absorption problems, and lead to immune alterations [12,21,26,27], thus generating conditions likely to trigger metabolic imbalances as well as inflammatory processes. In reality, there is a bidirectional interplay between RT and the microbiota, in which RT may cause microbiota dysbiosis. Yet, these microbiota alterations may be important determinants of RT anti-tumor effectiveness [28,29].

This bidirectional interplay between RT and the microbiota becomes further complicated when antibiotics (ABTs) are included at some point during the treatment of cancer patients. The problem is that, while some ABTs have been recognized as anti-cancer agents based on their anti-proliferative and/or pro-apoptotic activity on cancer cells, they can also act as pro-tumorigenic factors by their ability to cause substantial qualitative and quantitative modifications of the gut microbiota, consequently reducing the efficacy of anti-cancer drugs and/or increasing the toxicity responses to cancer treatments [30,31]. In some cases, ABTs have been reported to diminish the viability and clonal expansion of cancer stem cells (CSC) and, consequently, decrease the incidence of tumor recurrence, resistance to therapy, and even the establishment of distant metastases [32]. Yet, in other instances, ABT therapy has been reported to negatively impact the outcome of different anti-cancer therapy modalities against various tumor types, including the use of immune checkpoint inhibitors [33,34,35,36], chemotherapeutic drugs [37,38], or chemoradiation (CRT) protocols [39], particularly when one or more cycles of ABTs were applied before the anti-cancer therapies [34,39]. Given the circumstances, the possibility of using ABTs in combination with other anti-cancer therapies remains somewhat controversial. The situation is even more unclear in the case of RT, as the number of RT-focused studies exploring the effect of ABTs on RT efficacy and RT-derived adverse events is smaller than those reported for other anti-cancer therapies. In this review, we examine currently available data on this issue and discuss possible alternatives to minimize the potential adverse effects of ABTs on the microbiota and RT outcomes.

## 2. Radiation Therapy Affects the Human Microbiota

### 2.1. Radiation Therapy Causes Gut Microbiota Dysbiosis

The gut microbiota has been linked to many beneficial functions for the health of the host [40]. Alterations of the gut microbiota resulting in overt dysbiosis can disrupt the proper functioning of various physiological determinants of host health. While exploring the role that RT, alone or in combination with chemotherapy, plays in causing gut microbiota dysbiosis, one finds that, similarly to other cancer treatment modalities, RT causes its own specific challenging short- and long-term secondary effects on the patients, and that some of these changes are likely derived from the effects that IR has on compositional quantitative and qualitative changes of the gut microbiota [22]. Exposure to radiation damages the IEB, which, in addition to negatively affecting essential intestinal nutrient absorption functions, compromises the barrier function itself as the damaged IEB may allow the translocation of bacteria deeper into the body and facilitate bacterial metabolites access to the blood circulation [41]. The IEB damage and the abnormal movement of intestinal bacteria can lead to widespread cytokine-induced inflammatory reactions, which affect a variety of processes, thereby resulting in clinically noticeable secondary effects [42,43]. The fact that radiation exposure causes inflammation was confirmed by the increased levels of systemic inflammation markers such as interleukin (IL)-1β, IL-6, and tumor necrosis factor-α (TNF-α) detected after irradiation both in patients and in mouse models [27,44,45]. Moreover, it was shown that patients with higher levels of systemic inflammation markers also experienced worse adverse effects post irradiation than patients with normal levels of inflammation markers [45].

As indicated above, in addition to the pro-inflammatory effects, radiation exposure can also cause the dislocation of gut bacteria [46] and lead to the formation of reactive oxygen species (ROS) through the radiolysis of water [12,22,47,48]. ROS are highly reactive due to their unpaired electrons in the valence shell and can cause damage to the DNA and other cellular structures of microbes in the gut microbiota leading to changes in its composition [49,50]. Studies in which patients and mice were subjected to various radiation doses either alone or combined with chemotherapy consistently showed decreased overall gut microbial diversity following RT exposure [42,51,52]. Additionally, the application of methods for diversity measurements [53], which highlight the change or differences in the composition of the microbiota, showed that RT caused a decrease in alpha diversity (microbial variety in a single sample) and an increase in beta diversity (variation between microbial communities) of the gut microbiota [44,45,54]. These observed changes in alpha and beta diversity correlated with the progression of the radiation-induced injuries. In patients subjected to pelvic RT, those who progressed to radiation enteritis (RE) had a reduced alpha diversity and increased beta diversity compared to patients who did not progress to RE after pelvic irradiation [45]. RE leads to common irradiation symptoms such as diarrhea, nausea, vomiting, and abdominal cramps [45,55,56].

A common finding in most studies (Table 1) was the alteration in the ratio of *Firmicutes* to *Bacteroidetes* after RT. There was a decrease in the abundance of *Firmicutes* concomitant with an increase in abundance of *Bacteroidetes* [3,44]. *Bacteroidetes* and *Firmicutes* are dominant phyla in the microbiota of the healthy human gut [57,58]. The ratio of the two is often considered to be a marker of host health [40], as previous studies reported that a lower ratio of *Firmicutes* to *Bacteroidetes* was consistently found in patients who developed adverse symptoms post irradiation in comparison with patients with no apparent secondary effects [42,54].

RT has been found to reduce the relative abundance of healthy gut bacteria while allowing the growth of potentially pathogenic bacteria. The relative increase in members of the phylum Verrucomicrobia was identified in samples of the irradiated large and small intestine of mice using 16S rRNA gene sequencing. Verrucomicrobia is commonly found in increased abundance due to dysbiosis caused by broad-spectrum antibiotic treatment. Conversely, the genera *Prevotella* and *Mucispirillum* were found in lower abundance in irradiated large intestine samples. These two genera regulate cellular functions related to the development of innate immunity by the mucosa-associated microbiota and the expression by the host of genes encoding toll-like receptors and enzymes involved in purine metabolism and mucin oligosaccharide degradation [59]. The inflammatory environment created by RT promotes the growth of pathogenic pro-inflammatory bacteria while suppressing the growth of anti-inflammatory bacteria. These changes in microbial abundance further aggravate the inflammation process. CRT of rectal cancer patients increased suspected pro-inflammatory members of the phylum Bacteroidetes and the genera *Bacteroides* and *Escherichia*, such as *B. fragilis* and the adherent invasive (AIEC) and diffusely adherent (DAEC) strains of *E. coli* [54,57]. These findings were also significant in patients experiencing fatigue post RT compared to non-fatigued patients. Other studies showed a rise in pro-inflammatory bacteria belonging to genus *Sutterella* (*S. wadsworthensis* and *S. parvirubra*) and decreased proportions of anti-inflammatory bacteria such as *Faecalibacterium prausnitzii* and *Prevotella histicola*, and additional members of the Firmicutes phylum, such as *Lachnospira pectinoschiza*, *Roseburia intestinalis*, and others [27,60,61,62,63].

Changes in the microbiota after RT can also result from compensatory dynamics and/or relative microbial differences in radiosensitivity. In this regard, the abundance of *Akkermansia* spp., which is known to aid in mucus degradation, was increased after irradiation [59]. On the other hand, although *Bifidobacterium* and *Lactobacillus* are known probiotics, the abundance of *Bifidobacterium* increased post irradiation while the abundance of *Lactobacillus* decreased. This different behavior could be due to the presence of oxygen radical-scavenging enzymes such as catalase and superoxide dismutase in *Bifidobacterium* that support a radioresistant phenotype. The lack, or much lower levels, of such enzymes in *Lactobacillus* could explain the reduced abundance of *Lactobacillus* after irradiation [43].

Although many studies have reported the induction of dysbiosis after irradiation, there are cases in which the overall levels of diversity did not seem to be affected. In this regard, a study on mouse intestinal microbiota after RT showed no significant difference in intestinal microbiota diversity when comparing irradiated samples to controls. However, the results from this study agreed with the specific changes in abundance seen at the phylum and genus level described by reports mentioned above [59]. Interestingly, a study comparing the gut microbiotas of fatigued and non-fatigued patients after treatment with CRT showed a significant increase in the abundance of anti-inflammatory bacteria of the *Faecalibacterium* genus in patients that developed fatigue symptoms post irradiation [54]. The apparent discrepancy between these findings and data from other reports mentioned above might be due to smaller sample sizes and/or differences in microbiota analysis methods, radiation doses, and the use of radiation alone or in combination with chemotherapy. These contrasting findings bring to light the difficulty of standardization while assessing the effect of RT on the human microbiota. This is a critical issue that must be addressed in the future in order to obtain meaningful information.

### 2.2. Radiation Therapy Causes Oral Microbiota Dysbiosis

The human body maintains symbiotic interactions with microbes housed in anatomical locations beyond the gut. Therefore, similar to the effects observed in the gut, the use of RT for the treatment of head and neck cancer (HNC) patients also leads to disruptions in the oral microbiota. In the case of the oral microbiota, it has been postulated that one of the reasons for the induction of dysbiosis is the observed reduction in salivary flow. RT directly damages the salivary glands in HNC patients, leading to hyposalivation and caries development. The RT-induced changes in the oral microbiota can further aggravate the caries situation [64,65,66]. A study on HNC patients undergoing RT showed that, among irradiated patients, the relative abundance of *Prevotella melaninogenica* decreased in patients that did not develop caries. *P. melaninogenica* is associated with caries in young children [67]. Conversely, the microbe *Abiotrophia defectiva*, associated with a healthy microbiota, was found at a lower relative abundance in patients who developed caries after irradiation [65].

RT can also lead to oral mucositis (OM) due to the pro-inflammatory environment created by radiation leading to erythema, edema, and mucosal ulcerations in the oral mucosa, tongue, and pharynx [68,69]. OM leads to poor quality of life, increased use of narcotic analgesics, needs for assisted nutritional support, and changes in dose, fractionation, and intervals in cancer RT protocols [69]. The process of RT leading to OM has been associated with oral microbiota dysbiosis caused by irradiation. As seen with cases of dysbiosis in the gut microbiota, RT can also lead to the early expression of inflammatory markers such as TNFα, NF-κB, and IL-1β [70], and the early creation of such an inflammatory environment can cause OM either directly or indirectly by disrupting the oral microbiota, which in turn amplifies inflammation and thereby OM in the oral cavity [71]. The disruption to the oral microbiota leads to a decrease in health-associated species, allowing for the growth of potentially pathogenic species [72]. Of these pathogenic species, the most significant to the oral microbiota are Gram-negative anaerobes associated with periodontitis and mucositis [66,71,73]. Comparative analysis of the microbiota in samples of oral swabs (buccal mucosa and lateral tongue) collected post RT from patients with grade ≥ 2 OM relative to patients with grade ≤ 1 OM (following the World Health Organization’s OM Assessment Scale) showed a higher abundance of Gram-negative anaerobic genera such as *Tannerella*, *Sneathia*, *Mycoplasma*, *Capnocytophaga*, *Bacteroidales G2*, *Porphyromonas*, and *Eikenella*. Of particular interest are *Tannerella* and *Porphyromonas* species that have been associated with periodontitis [66]. Another study collecting post-irradiation retropharyngeal mucosal samples found an increase in the abundance of genera *Actinobacillus*, *Mannheimia*, *Streptobacillus*, and unclassified *Pasteurellales* in early stages of OM in patients that later developed severe OM (grades 3–4 in the scale of the Radiation Therapy Oncology Group, RTOG) as compared to patients with mild OM cases [73]. The involvement of these prominent Gram-negative anaerobic species in OM development has been associated with their ability to amplify the process of oral mucosal inflammation [74]. Research on the changes in alpha diversity observed in the oral microbiota falls short on consistency compared to data on the alpha-diversity modifications of the gut microbiota. This may be the case because different combination therapies are typically used along with RT to treat patients with HNC and colorectal carcinoma (CRC) [71,73,75,76].

## 3. Human Microbiota Affects Radiation Therapy Outcomes

### 3.1. Dysbiosis Influences the Adverse Effects of Radiation Therapy

As described above, RT has profound effects on the human microbiota. Still, the complexity of the interaction does not end there, as the relationship between RT and the human microbiota is a reciprocal one. Once RT causes changes to the microbiota, these alterations, in turn, influence the outcomes of radiation treatment. The microbiota dysbiosis modifies the effects of RT on the tumor and the adjacent tissue and, consequently, the side effects due to radiation exposure [68,70,71]. Fecal microbiota transplantation (FMT) to wild-type mice from mice that, after undergoing irradiation, developed colon inflammation, rendered the recipient mice susceptible to dextran sodium sulfate-induced colitis and radiation injury. Additionally, in vitro incubation of normal colonic epithelial cells with fecal samples from irradiated mice increased the production of pro-inflammatory factors, namely, TNF-α and IL-1β [50] by the treated cells. A study examining the changes in gut microbiota after irradiation and their relation to cervical cancer patients that develop RE showed increased inflammation and, accordingly, a rise in pro-inflammatory factors. Moreover, bacterial samples from patients who did not develop RE were enriched with *Bacteroides*, *Bacteroidaceae*, and *Plebeius*. In contrast, samples from patients who did develop RE were enriched with *Megamonas*, *Novosphingobium*, and *Prevotella*. When these bacterial samples from patients were co-cultured in vitro with human normal colonic epithelial cells, there was an increase in TNF-α and IL-1β secretion when compared to non-RE patients. Furthermore, IEB markers such as the tight junction proteins ZO-1 and occludin were significantly down-regulated in RE patient bacterial sample co-cultures compared to the co-cultures established with non-RE patient samples [45]. Similarly, FMT from mice undergoing radiation to GF mice resulted in the induction of IL-1β secretion in vivo [12]. These results, along with the information presented above, further support the notion that the microbiota dysbiosis mediated by radiation-induced inflammation further aggravates the inflammatory process and, consequently, may affect the radiation treatment outcome.

The pre-existing peculiarities of the gut microbiota of any given patient can also change the outcome of the RT treatment of that individual. Therefore, characterizing these pre-existing gut microbiota features can serve as a tool to predict the RT outcomes [74,75]. Patients who had diarrhea after undergoing pelvic RT were shown to have a higher *Firmicutes* to *Bacteroidetes* ratio and a lower microbial alpha diversity before therapy compared to the pre-existing microbiota of patients who did not develop diarrhea. The pre-existing microbiota of patients who developed diarrhea included a higher proportion of *Bacteroides*, *Dialister*, *Veillonella*, and a decreased proportion of *Clostridium* clusters XI and XVIII, *Faecalibacterium*, *Oscillibacter*, *Parabacteroides*, and *Prevotella* [74]. In contrast, the microbiota of patients with co-occurring adverse effects at the end of CRT for rectal cancer included a higher proportion of *Bacteroides* before the CRT treatment. Conversely, patients who did not develop co-occurring symptoms had a higher proportion of gut microbes belonging to the genus *Clostridium*, the *Ruminococcaceae* family, and members of the *Lactobacillaceae* family prior to CRT [30,42,77,78,79]. Butyrate-producing bacteria can break down polysaccharides through anaerobic fermentation to produce SCFAs, which are important in inhibiting cancer cell growth [45,57]. SCFAs and tryptophan metabolites also reduce pro-inflammatory cytokines and promote the secretion of anti-inflammatory cytokines, which contribute to restraining radiation-induced damage [80,81]. These data on the importance of the particular features of the patients’ pre-existing microbiota point to the potential of using microbiota analysis in personalized treatment protocols to determine the outcome of RT in cancer treatment and possibly using the gut microbiota as a biodosimeter for RT treatment planning [42,52,56].

### 3.2. The Microbiota Plays a Role in Enhancing Radiation Therapy

Traditional cancer therapies have proven their efficacy in reducing tumor growth and, at times, eliminating tumors. However, these conventional forms of therapy encounter roadblocks with certain tumor types. In the case of radiation, an oxygenated environment is needed to maximize RT effectiveness. Therefore, in some cases, hypoxia and the low pH conditions allow tumors to acquire radiation resistance [82,83]. Therefore, traditional RT may require the combination with supplementary forms of therapy to improve cancer prognosis. The use of anaerobic bacteria has been identified as one possible supplemental therapeutic modality because anaerobic bacteria can colonize all tumor areas regardless of their oxygenation state and help destroy even the hypoxic tumor regions [84]. In a study using the commensal *Bifidobacterium infantis*, administration of *B. infantis* to mice, administration of a *B. infantis*-specific monoclonal antibody plus RT allowed for more significant inhibition of tumor cell proliferation, a reduction in the blood supply to the tumors, and a more substantial tumor cell apoptosis when compared to the use of RT alone. Mice treated with the combination therapy also had more prolonged survival than mice given RT alone or *B. infantis* antibody alone [85]. A similar study was conducted to test the synergistic interaction of *Lactobacillus rhamnosus* (ATCC 7469) exopolysaccharides (EPS) with low-level IR in halting CRC progression in rat models. EPS were used based on their ability to form a protective cover for probiotics [86]. This study showed that using *L. rhamnosus* in combination with low-level IR improved modulation of signaling growth factors involved in inflammation, which contributed to slowing down CRC progression compared to animals treated with *L. rhamnosus* alone, IR alone, or the untreated controls [87]. The use of bacterial strains as supplements to traditional RT seems very promising. It is noteworthy that the combination of RT with bacterial supplements allows for a better prognosis, possibly attainable even with lower doses of radiation, which will make RT more effective and simultaneously reduce patients’ adverse effects.

## 4. Antibiotics Affect the Radiation Therapy-Microbiota Interaction

### 4.1. Antibiotics Affect the Composition of the Human Microbiota

Many studies focused on the effects of ABTs on cancer treatment because some ABTs have been described as being able to slow cancer cell proliferation, promote their apoptotic cell death, or have anti-epithelial-to-mesenchymal-transition (EMT) activity [30]. However, there are conflicting research data on the effects that ABTs have on cancer therapy through their modulation of the microbiota. ABTs are the most commonly used agents directly affecting the human microbiota. Treatment with ABTs can alter the composition of the microbiota and, therefore, affect the outcome of RT. In this regard, a study with mice on the effects of treatment with ABTs before RT found that ABT pretreatment can, to a certain extent, reverse the microbiota dysbiosis and the intestinal damage caused by RT [77]. This study administered a cocktail of vancomycin, ampicillin, and gentamicin to the mice before radiation exposure. The experimental group in which ABTs were administered showed a recovery of radiation-induced intestinal damage, a process that seemed to be associated with the ability of the ABT cocktail to regulate LPS/TLR4/MyD88/NF-κB p65/macrophage polarization/TGF-β1/Smad-3 signaling pathways. Consistent with other studies, abdominal radiation caused a disorder in the gut microbiota and a decrease in intestinal microbiota diversity. RT led to an increase in the proportion of members of pathogenic phyla in the microbiota and a concomitant reduction in the abundance of microbes belonging to beneficial phyla. The experimental group that received ABT pretreatment also showed a better reconstitution of the intestinal microbiota mirroring its pretreatment composition than the radiation-only group. Furthermore, the ABT pretreatment group had a higher relative abundance of Verrucomicrobia, which is associated with anti-inflammatory functions.

Radiation exposure leads to the production of ROS and reactive nitrogen species (RNS) and the leakage of electrons from mitochondria. Similar to the damage on bacterial DNA leading to dysbiosis, the damage done by RT at the molecular level to intestinal structures manifests as disruption of tight junctions and induction of cell death in the crypt and villi epithelia. This disruption can lead to inflammation and IEB destruction, ultimately causing the leakage of intestinal content. In particular, the leakage of bacteria and fungi can lead to further systemic inflammation [77]. Therefore, the contribution of ABTs toward repairing the IEB and reconstituting the intestinal microbiota targets the root of the problems that manifest as RT side effects. Contrastingly, research on a combination of ampicillin, imipenem, cilastatin, and vancomycin showed that the ABTs hurt commensal bacteria and, in turn, the anti-tumor response to RT [78]. Administration of the ABT combination before RT led to a failure of the radiation treatment to delay tumor growth. Microbiota analyses showed that ABT pretreatment decreased bacterial diversity and increased fungal diversity. Specifically, the ABT treatment reduced the alpha diversity of bacteria. There was a significant decrease in the order of Clostridiales and a more prominent presence of orders of Lactobacillales and Burkholderiales. Additionally, the fungal order of Saccharomycetales, mainly represented by the yeast *Candida albicans*, increased in abundance. *C. albicans* was found to reduce the tumor growth delay and shortened the survival time following RT [78]. Commensal bacteria were found to aid in generating activated T cells after RT. In contrast, commensal fungi species appear to modulate the immunosuppressive tumor microenvironment through their combined effects on macrophages and T cells. The antifungal treatment had a more beneficial effect when used before RT [78].

### 4.2. Antibiotics Affect Radiation Therapy Efficacy

Over the last 10–15 years, the scientific community has witnessed the slow but steady emergence of a research area focusing on repurposing drugs that already had FDA approval to be used to treat diseases other than the ones they were initially approved for [88]. An important objective of this type of strategy has been the repurposing drugs to treat cancer [89,90,91,92]. Most importantly, in agreement with similarities identified between cancer cells and infectious microorganisms [93], some of the repurposed drugs identified as having significant anti-cancer activity belong to several classes of antimicrobial agents [94,95]. In addition, and from a more relevant point of view for this review, several antimicrobial agents have been identified as having radiosensitizing effects in addition to their anti-tumor properties against various types of tumors [92,95]. Performance of in culture and preclinical studies with breast cancer and melanoma models demonstrated that ABTs such as cefepime, cephalosporins, and doxycycline [79,96] and antifungals such as terbinafine and clotrimazole [97] not only had anti-cancer activity but all of them also enhanced the effect of RT in combination protocols [95]. Among the radiosensitizing ABTs, doxycycline is perhaps the best understood from a mechanistic point of view. Doxycycline, an ABT known to inhibit bacterial protein synthesis by interacting with the 16 S rRNA, is typically used to treat acne, and it is almost completely absorbed orally with a serum half-life of 18 to 22 h, at a dose of 200 mg per day. Pretreatment with doxycycline prior to RT of breast tumors resulted in a 4.5-fold increase in the radiosensitivity of the CSCs. The increase in sensitivity was mediated by the ability of the ABT to down-regulate the expression of the DNA-PK protein, an enzyme required for proper DNA repair by nearly 15-fold [79,88]. The radiosensitizing effect of certain antimicrobial drugs allows reaching the desired tumor reduction/regression outcome with a lower IR dose than that required otherwise and, consequently, reduces the short- and long-term adverse effect of IR exposure for patients undergoing RT. Nevertheless, research has also been conducted on identifying ABTs that may be used by themselves or in combination with other agents to mitigate further IR side effects, not only in the context of RT but also in cases of unexpected, accidental IR exposure in the absence of cancer. In this regard, the ABT enrofloxacin, combined with the angiotensin-converting enzyme (ACE) inhibitor lisinopril, can effectively counter acute radiation syndrome by mitigating early and late radiation injuries [80,98,99].

## 5. Interventions to Improve the Efficacy of Radiation Therapy

### 5.1. Probiotics Use as an Intervention to Radiation Toxicity

Based on the scientific reports described above, it seems apparent that microbial communities present in different parts of the human body play a significant role in determining the radiation treatment outcomes for various types of cancer. Therefore, it is worthwhile looking into bacterial interventions that may help reduce the adverse effects of RT. The most enterprising of such interventions involve the use of probiotics, which most often include bacterial species commonly found in the human body. The mainstay probiotics currently used are *Lactobacillus* and *Bifidobacterium* species [49,81,82,83]. Probiotics were reported to lessen RT side effects by modulating the immune system, down-regulating pro-inflammatory cytokines, modulating apoptosis, and reversing dysbiosis [83]. The administration of *L. rhamnosus* GG (LGG) in mouse models subjected to irradiation in vivo and in vitro showed that the radioprotective properties of LGG toward intestinal epithelium were mediated through the continuous release of lipoteichoic acid (LTA). When LGG was administered after irradiation, LGG released its biologically active component LTA, which binds to the TLR2 toll-receptors present on macrophages. This binding resulted in macrophage release of the chemokine CXCL12, which, in turn, binds to its CXCR4 receptor on mesenchymal stem cells (MSCs) expressing COX-2. MSCs then migrate from the lamina propria of the villus to the pericryptal lamina propria, where COX-2 promotes the release of prostaglandin PGE2. PGE2 has anti-apoptotic effects on the adjacent epithelial stem cells, blocking radiation-induced epithelial stem cell apoptosis. These findings showed that the cascade of immune responses caused by LTA protects normal tissue in the small intestines from radiation injury. A shortcoming was that the MSCs expressing COX-2 are only found in the GI tract, and PGE2 has a short half-life, allowing it only to affect cells located close by [85]. Therefore, LGG cannot extend its radioprotective effects to other regions such as the bone marrow during RT. Although this research was performed on mouse models, LGG is currently undergoing clinical trials (NCT01790035) to establish its efficacy as a radioprotectant for patients receiving RT.

Research focusing on changes in the severity of RT side effects after probiotic interventions showed a decrease in diarrhea, vomiting, and reverting to normal stool consistency [82,83,86]. A study on the effects of probiotics with honey, which has antioxidant and anti-inflammatory properties, showed that patients who were given probiotics or probiotics plus honey had normal stool consistency and required fewer antidiarrheal medications after RT than those given placebo [86,87]. However, the administration of probiotics alone and probiotics plus honey resulted in bloating compared to the controls [82]. Studies on *Lactobacillus* and *Bifidobacteria* showed that administration after or before RT reduced the incidence of diarrhea and the use of antidiarrheal medication (loperamide) and resulted in a significant weight gain [83,100]. LGG was also found to be as effective as aminosalicylic acid (5-ASA), a standard treatment for proctitis, the inflammation of the rectum lining, in controlling the clinical and histopathological symptoms caused by radiation exposure in rat models [100,101].

Probiotics can reduce RT adverse effects by reducing the expression of pro-inflammatory markers. Administration of *Bacillus licheniformis* (ZCS) reduces inflammation by reversing the enhancement of pro-inflammatory factors, ET, CRP, TNF-α, IL-1β, and IL-6, induced by RT [85]. Probiotics can also modulate the immune system by increasing the number of immune cells that were previously down-regulated by concurrent chemoradiation therapy (CCRT). Results from a study on the effect of probiotics in reducing the severity of OM due to CCRT showed that probiotics (containing *Bifidobacterium longum*, *Lactobacillus lactis*, and *Enterococcus faecium*) were able to reverse the decrease in the number of CD4+ T cells, CD8+ T cells and CD3+ T cells caused by CCRT [102]. CD3+ T cells play an essential role in cell-mediated immunity, while CD 8+ T cells can mount the immune response against pathogens, tumor cells, infected cells, and damaged cells [103,104].

The use of probiotics toward improving RT anti-tumor outcomes is a real possibility, but studies still provide conflicting information on the efficacy of probiotics for the management of RT-induced adverse events. This problem may be due to differences in the bacterial strains used in the various studies. While a study on a probiotic combination containing *B. longum*, *L. lactis*, and *E. faecium* showed that probiotics could not help with weight gain post CRT, another survey of probiotics containing *L. rhamnosus* GG showed an increase in weight gain with probiotic administration post RT [100,102]. Furthermore, a systematic review on the effect of probiotics on GI symptoms induced by pelvic RT showed little evidence that probiotics can reduce diarrhea during or at the end of RT [105]. Future research efforts need to use the same *Lactobacillus* and *Bifidobacterium* genera to standardize their findings. A limitation to the use of probiotics is the possibility of causing bacteremia. Although none of the human studies described in this review reported that probiotic administration led to bacteremia, cancer patients receiving myelosuppressive chemotherapeutic agents should be closely monitored when receiving probiotics.

### 5.2. Prophylactic Agents for Reversing Radiation-Induced Dysbiosis

Prophylactic agents other than probiotics are also being evaluated for the potential to alleviate symptoms of RT without the danger of bacteremia. These agents are relevant due to their ability to suppress pathogenic bacteria while promoting the growth of beneficial microbes. One such agent is VND3207, a derivative of vanillin found in vanilla, reported exerting radioprotective effects without any significant cytotoxicity. Pretreatment with VND3207 in mouse models improves the mice’s survival rate and alleviates radiation-induced weight loss post irradiation. VND3207 reversed the increased relative abundance of *Lachnospiraceae* NK4A136 group and *Desulfovibrio* genera and the decreased relative abundance of *Bacteroides* and *Ruminococcaceae* UGC-014 caused by exposure to 9 Gy total body irradiation [106]. *Lachnospiriaceae* and *Desulfovibrio* have been researched for their pathogenicity. *Lachnospiriaceae* are associated with inflammation, while *Desulfovibrio* has been linked to non-communicable diseases such as ulcerative colitis, colon cancer, and the metabolic alterations seen in obesity and insulin resistance [107,108,109,110,111,112]. On the other hand, *Bacteroides* were seen to have a beneficial relationship when found in the human gut [113]. Lastly, VND3207 restored the *Firmicutes* to *Bacteroidetes* ratio, associated with healthy gut states [106]. The Guiqi Baizhu Decoction (GQBZD) used in Chinese traditional medicine and phycocyanin (PC), a protein responsible for photosynthesis in Spirulina (a mixture of three blue-green algae of the *Arthrospira* genus), are other potential prophylactic agents that have been evaluated for their potential to reverse the microbiota dysbiosis caused by RT [114,115]. GQBZD was able to increase gut microbial diversity post irradiation and reverse changes in the abundance of *Firmicutes*, *Bacteroidetes*, and *Proteobacteria*, and the ratio of *Firmicutes* to *Bacteroidetes* [114]. Similarly, PC promoted the growth of beneficial bacteria such as those related to the synthesis of SCFAs and suppressed the growth of pathogenic strains that proliferated post irradiation [115].

## 6. Conclusions and Future Perspectives

RT has been an effective modality of traditional cancer therapy for decades. In many cases where surgical removal of a tumor is not possible, RT is the mainstay treatment. However, RT causes various adverse effects such as caries, oral ulceration, diarrhea, cachexia, fatigue, vomiting, and others, depending on the site of radiation and the treatment plan used. These side effects limit how RT can be used as an effective cancer treatment. The recent focus on the host-microbiota crosstalk has shed some light on the role of the microbiota in cancer progression and therapy. More specifically, a widening body of literature looks into the disruptions of the microbiota caused by irradiation and the role that radiation-induced dysbiosis plays in developing RT side effects. Radiation exposure can: (a) cause cell structure damage in microbes through the formation of ROS, (b) disrupt the mucosal epithelial barrier through inflammation leading to translocation of bacteria, (c) cause hyposalivation disrupting the oral microbiota, and (d) create a pro-inflammatory environment that leads to dysbiosis. The last mechanism is of particular interest as it speaks to the reciprocal relationship between RT and the human microbiota in determining the outcome of cancer therapy. RT leads to a pro-inflammatory environment that causes and is further aggravated by dysbiosis of the human microbiota. These two factors (inflammation and dysbiosis) play a primary role in determining the extent of side effects from RT and the anti-cancer treatment outcome.

Due to the integral role that microbiota dysbiosis plays in cancer prognosis, interventions to control/prevent dysbiosis should be a primary focus in future research. Interventions before RT such as FMT to induce patient-specific microbiota alterations [116] and implementation of nutritional strategies that may support a healthy intestinal mucosa [117] may contribute to improving RT outcomes. In addition, the use of ABTs to modulate the microbiota and improve RT efficacy is a promising field of study. ABTs represent a cost-effective treatment based on compounds that already exist in the market. Specifically, the use of ABTs already approved by the U.S. Food and Drug Administration can help ease the transition toward the use of ABTs as RT supplements. Additionally, probiotics, mainly *Lactobacillus* and *Bifidobacterium* species, are of particular interest in tackling microbiota disruptions [118]. It is important to note that a focus on genus rather than species can be a problem due to potential pathogenic species in the genus and the inability to standardize data when varying species are used in different research trials. Therefore, focusing on higher-quality genetic sequencing, identifying specific species for probiotic use, and identifying specific species in dysbiosis research studies can help draw a better picture of the problem and its solution. The use of other prophylactic agents is also worthwhile. The side effects due to RT are exacerbated by an increased abundance of pro-inflammatory bacteria concomitantly with a decrease in anti-inflammatory bacteria. Because of this, prophylactic agents with the potential of reversing these changes in the relative abundance of bacterial communities can help relieve the side effects, thus allowing for better RT outcomes. The human microbiota and the use of modulatory ABTs are worthy fields of research in cancer therapy, as they will allow a better mechanistic understanding of the development of RT adverse effects and potentially lead to workable solutions. Future research with more standardized approaches in terms of radiation doses, identification of microbes down to the species, and better characterization of the peculiarities of the pretreatment microbiota can help harness the full potential of modulating the human microbiota to consistently improve cancer treatment outcomes. In this regard, newly developed methodologies [119] may facilitate performing personalized microbiota screenings [120] on patients before radiation therapy and provide accurate information that will become extremely useful to optimize all aspects of radiation treatment planning.

## Figures and Tables

**Table 1:** Radiation therapy-induced changes in the gut microbiota.

Study Aims	Radiation Dose	Chemotherapy	Microbial Changes to Radiation	References
Correlation between fatigue, diarrhea and gut microbial changes during pelvic RT	Total dose of 44–50 Gy as 1.8–2.0 Gy/day 5 times a week for 5 weeks	No	Before RT, genera *Alistipes*, *Bacteroides*, *Barnesiella*, *Oscillibacter*, *Parabacteroides*, *Prevotella*, and *Ruminococcus* were less abundant, and genera *Faecalibacterium*, *Clostridium XI*, *Roseburia*, and *Veillonellain* were more abundant in cancer patients compared to healthy subjects	[44]
			*Firmicutes* to *Bacteroidetes* ratio decreased	
			Genera *Bacteroides* and *Clostridium_XIVa* were more abundant, and genera *Faecalibacterium*, *Lachnospiracea*, *Oscillibacter*, *Roseburia*, and *Streptococcus* were less abundant after RT	
Relation of alterations in gut microbiota to enteritis in patients receiving pelvic radiation therapy	Total dose of 50.4 Gy in 1.8 Gy fractions	No	Patients who developed radiation enteritis (RE) had a lower relative abundance of phylum *Bacteroidetes* and higher abundance of genus *Serratia*, *Bacteroides*, and *Prevotella_9* than non-RE patients	[45]
Effects of RT on the microbiota composition of large and small intestines	Single 8 Gy dose	No	Phylum *Verrucomicrobia* were found in large and small intestines of irradiated mice but not in controls	[55]
			Phylum *Bacteriodetes* and *Proteobacteria* were more abundant, while phylum *Firmicutes* and *Actinobacteria* were less abundant in the large and small intestines of irradiated mice when compared to controls	
			Mice irradiated in the large intestines had a large abundance of genera *Alistipes*, *Lactobacillus*, and *Akkermansia*, but reduced abundance of genera *Barnesiella*, *Prevotella*, *Bacteroides*, *Oscillibacter*, *Pseudoflavonifractor*, and *Mucispirillum*	
			In the small intestines, irradiation caused an increase in genus *Corynebacterium* and a decrease in genus *Alistipes*	
Evaluation of: (a) relation of gut dysbiosis to the onset of adverse symptoms (fatigue, sleep disturbances, and depression) caused by CRT; and (b) machine learning to predict if patients will have symptoms on the basis of features of their gut microbiota	45–50 Gy in 25–28 fractions to the pelvis	Continuous infusion of 5-FU (225 mg/m^2^ over 24 h) or oral capecitabine (825 mg/m^2^ twice a day)	Patients with co-occurring secondary effects had a higher proportion of genus *Bacteroides* before chemoradiation (CRT) and a higher proportion of *Blautia2* at the end of CRT compared to patients with no adverse symptoms	[42]
			Patients with no secondary effects had a higher proportion of genera *Lactobacillus*, *Ruminococcaceae*, and *Lachnospiraceae* at the start of CRT and a higher proportion of *Bacteroides*, *Blautia1*, *Ruminococcaceae*, *Oscillibacter*, and Lactobacillus at the end of CRT	
Short-term changes in *Bifidobacterium* and *Lactobacillus* abundance after X-ray irradiation	2 Gy and 4 Gy	No	Increase in genus *Bifidobacterium* and decrease in *Lactobacillus* post irradiation in mice	[43]
Changes in gut microbiota during CRT and their relation to fatigue symptoms	45 Gy in 25 fractions and 6 to 8 Gy boosts in 3 or 4 fractions	Continuous infusion of 5-FU 225 mg/m^2^ over 24 h or oral capecitabine 825 mg/m^2^ twice a day for 5 days per week for 5 weeks	Patients receiving CRT that presented with fatigue after treatment had a higher abundance of *Proteobacteria* from the *Escherichia* genus, genera *of the* phylum *Bacteroidetes*, and *Faecalibacterium*, as compared to non-fatigued patients post CRT	[54]
Alterations of the rectal and fecal microbiota in patients with locally advanced rectal cancer undergoing neoadjuvant concurrent chemoradiation therapy (nCCRT)	45–50 Gy in 1.8–2.0 Gy daily fractions	Oral capecitabine	Genera *Porphyromona*, *Parvimona*, *Peptostreptococcus*, *Fusobacterium*, *Ezakiella*, and unidentified *Clostridiales* decreased in abundance, and *Lactobacillus* and *Streptococcus* increased in abundance in patients receiving nCCRT	[51]
Meta-taxonomy of the mucosal microbiota in patients undergoing neoadjuvant long course CRT for rectal cancer	45 Gy in 25 fractions over 35 days	Oral capecitabine (825 mg/m^2^ daily)	*Faecalibacterium* was in lower abundance in patients receiving radiation therapy	[58]
			*Lachnoanaerobaculum* was in lower abundance in patients receiving chemotherapy	
			*Allprevotella* was only found in patients receiving chemotherapy	
Effects of rectal RT on the microbiota, and their relation to tissue damage in mouse models	Four 550 cGy fractions with 24 h intervals between fractions	No	Radiation resulted in a lower abundance of *Firmicutes* and an increase in abundance of genera *Akkermansia*, *Bacteroides*, *Parabacteroides*, *Sutterella*, *Turicibacter*, and an unclassified genus belonging to the RF32 order	[27]
			Germ-free (GF) mice inoculated with fecal samples of mice exposed to radiation showed an increased abundance in phylum *Bacteroidetes* and *Proteobacteria* and a decreased abundance in *Firmicutes*	
			At the genus level, inoculated GF mice had increased proportions of *Suterella* and *Parabacteroides* and decreased proportions of members in the *Clostridiales* order	
Use of fecal microbiota as a biodosimeter of intestinal acute radiation injury	0, 4, 8, and 12 Gy	No	Fecal flora in rats after radiation exposure had decreased abundance of *Prevotella*, *Ruminococcus*, *Bifidobacterium*, and *Lactobacillus* and increased abundance of *Bacteroides* and *Enterobacterium* as compared to the control group (these changes were proportional to the radiation dose received)	[56]
Usefulness of changes in the gut microbiota for predicting nCCRT responses in LARC patients	50 Gy in 2Gy daily fractions	Capecitabine plus irinotecan	nCCRT caused an increase in *Lactobacillus* and *Streptococcus*; the rise in *Streptococcus* was exclusively in patients with tumor regression scores of 0 to 1	[52]

## Abbreviations

Antibiotics (ABTs); Cancer Stem Cells (CSCs); Chemoradiation (CRT); Colorectal Cancer (CRC); Concurrent CRT (CCRT); Epithelial-to-Mesenchymal Transition (EMT); Exopolysaccharides (EPS); Fecal Microbiota Transplantation (FMT); Germ-free (GF); Head and Neck Cancer (HNC); Intestinal Epithelial Barrier (IEB); Ionizing Radiation (IR); *Lactobacillus rhamnosus* GG (LGG); Lipoteichoic Acid (LTA); Mesenchymal Stem Cells (MSCs); Neoadjuvant CCRT (nCCRT); Neoadjuvant CRT (nCRT); Oral Mucositis (OM); Phycocyanin (PC); Radiation Enteritis (RE); Reactive Oxygen Species (ROS); Radiation Therapy (RT); Short-chain Fatty Acids (SCFAs).
